# Study on the dynamic response characteristics of lining structures in large-section tunnel blasting using JH-2 model analysis

**DOI:** 10.1038/s41598-024-60918-6

**Published:** 2024-05-07

**Authors:** Fengting Li, Ke Wu, Shengrui Li, Cao Wang, Yajun Liu, Zhongyu Dou

**Affiliations:** 1 School of Civil Engineering and Water Conservancy, Shandong University, Jinan, 250061 Shandong China; 2 Stecol Corporation Tianjin, Tianjin, 300384 China

**Keywords:** JH-2 model, Tunnel blasting, Lining structure, Dynamic response, Civil engineering, Computational science

## Abstract

The lining structures of tunnels are typically constructed using sprayed or cast concrete materials, and their performance and quality during tunnel excavation and blasting are crucial for the stability and safety of tunnels. Therefore, the safe distance between the lining structure and blasting source should be determined to avoid concrete damage caused by blasting vibrations. In this study, taking the subway tunnel of Danshan Station in Qingdao as an example, the JH-2 model is introduced as the constitutive model of the tunnel blasting simulation, and the JH-2 model parameters of the local surrounding rock are obtained by experiments, and finally the numerical simulation and theoretical verification are carried out to study the safety distance of shotcrete under various safety judgment standards. The results indicate that the JH-2 model can effectively simulate the propagation of stress waves under different media conditions, and the closer the strength parameters and pressure constant of the lining structure are to those of the surrounding rock, the safer the concrete–rock bonding interface. During tunnel blasting construction using the ring blasting method, the peak particle velocity (PPV) of the lining structure increases with an increase in the arch angle. Based on the numerical simulation results, we recommend that concrete lining be constructed at a distance of at least 62 m from the blasting source to avoid damage caused by vibrations. The effect of concrete tensile failure caused by longitudinal stress is much smaller than the damage to the bonding interface caused by the PPV and can be neglected.

## Introduction

In tunnel blasting operations, the surrounding rock walls exposed after blasting are typically protected and reinforced by lining structures, which are commonly used as important initial support structures to protect and strengthen the rock walls and form sturdy tunnel linings. However, in actual construction, blasting and lining support structures are usually conducted alternately to ensure project progress. The stress waves generated by blasting may damage the lining structures inside the tunnel, thereby affecting the initial support to the surrounding rock. Many scholars have analyzed the safety of lining structures through numerical simulations, among which the peak particle velocity (PPV) is an important factor for measuring the safety of lining structures.

Ahmed^1,2^ and other scholars have studied the impact of blasting on new and old lining structures during tunnel excavation by establishing numerical models. Guan^3^ used an explicit LS-DYNA algorithm and a fluid–structure coupling algorithm to simulate the vibration response and failure mode of lining structures under different loading weights and blasting distances. Tian^4^ used MATLAB for signal processing programming and analyzed the propagation rules of blasting vibrations in super-large cross-sectional shallow tunnels. The results showed that the maximum particle velocity on the ground decreased with decreasing distance and that the particle velocity in the vertical direction was greater than that in the horizontal direction. Yang^5^ evaluated the impact of explosion-induced vibrations on lining structures based on numerical simulations using the maximum tensile stress and Mohr–Coulomb criteria. The main failure mechanism of the lining structure was shear failure at the interface between the lining structure and rock, resulting in the loss of adhesion. Ahmed^6^ used three different modeling methods to study the support of lining structures in hard rock tunnels through numerical analysis. The stress response during the impact of a P-wave perpendicular to the lining structure–rock interface closest to the rock was simulated. Ming^7^ studied the failure modes and safe vibration velocities of new concrete linings under the action of explosion stress waves based on the Mohr–Coulomb, ultimate tensile stress, and ultimate tensile strain criteria. The results indicated that the incident stress and refracted waves in the lining had the greatest destructive effects. Feng^8^ derived theoretical formulas for calculating the relationship between the vibration velocity and stress of tunnel lining structures with small net distances under the propagation laws of explosive stress waves on different medium interfaces based on the stress wave propagation theory. Fangmei^9^ studied the destructive effects of blast-induced stress waves on the surrounding rock and lining structures of a tunnel using one-dimensional wave theory. The results showed that, for hard rock formations, the damage to the support structure was primarily determined by the tensile strength of the lining structure, whereas for soft rock formations, the damage to the support structure was primarily determined by the tensile strength of the interface between the lining structure and the surrounding rock. Zhao^10^ studied the dynamic response of lining structures of different ages under the coupling effect of explosive loads and initial stress transient unloading during tunnel excavation using the dynamic finite element method. The results indicated that the failure of the lining structures was primarily shear failure. Safe blasting distances of the concrete spraying layers at different ages were obtained. The Johnson–Holmquist ceramic (JH-1) constitutive model was proposed by Johnson and Holmquist^11^ to study the mechanical behavior of brittle materials. Based on the JH-1 model, a modified constitutive model called the Johnson–Holmquist-2 (JH-2) model was proposed^12^ and continuously optimized in subsequent studies^13–16^. Based on the above research, scholars Ma, Wei, Banadaki and He have used the JH-2 model to simulate the fracturing behavior of granite under a two-dimensional explosive action and compare the crack patterns with explosion experiments^17–20^. Wang^21^ adopted the JH-2 model as the constitutive model of rock materials in tunnel smooth blasting and proposed a fast and convenient method for determining JH-2 model parameters. The research results showed that a numerical simulation can effectively estimate the blasting damage of rocks and extract the PPV to estimate the damage range and degree of the tunnel-surrounding rock. Paweł^22^ determined the parameters of the JH-2 model for limestone based on experimental and literature data. The mesh density was simulated, and the results showed that some material parameters depend on the element size and should be adjusted according to the problem scale and geometric shape.

In summary, the existing research lacks a study on the dynamic response laws of large-section ring-blasting construction, and further expansion of the simulation of the JH-2 model in tunnel blasting is required. To address these problems, based on the Qingdao Danshan tunnel project, which involves the construction of a large-section tunnel using the ring blasting method, we employed a combination of numerical simulation and theoretical verification to study the safe stress and vibration velocity of different types of lining structures. The research findings can provide a basis for determining a safe lining distance in similar large-section ring-blasting tunnel projects.

## Project overview

The Dan Mountain Station of Qingdao Metro is a subway station constructed using a large-section tunnel structure. The station is located at the intersection of Heilongjiang Middle Road and Shimeian Road in Qingdao City. It is situated beneath Heilongjiang Road and arranged in a north–south direction. The station is a two-story island-type underground excavated station with a total length of 215 m and a standard section width of 21.1 m. The total construction area of the station is 16,877 m^2^. The station has three entrances/exits, two ventilation pavilions, one accessible elevator, and one emergency exit.

The rock formation at the tunnel site consists of moderately to slightly weathered granite. The overlying rock thickness ranges from 15.9 to 19.6 m. The initial support of the arch section of the tunnel is a single- or double-layer support. In the process of tunnel excavation, Danshan Station adopts ventilation shaft and inclined shaft as construction channels to enter the main structure of the station for construction, due to the complex structure and geological conditions of the station, the annular step method (annular blast method), the double-sidewall guide pit method, the step method and other methods are used in the project to combine the comprehensive construction, because the main structure in this project adopts the ring explosion method construction and the blasting surface is the largest, so this study analyzes the main ring explosion method.

In the Qingdao region, intrusive rocks are well-developed, with intrusion occurring during the Paleoproterozoic, Mesozoic, and Cenozoic eras. Among them, the Jiaonan period in the Paleoproterozoic and the Yanshan period in the Mesozoic are the main intrusions, with a widespread distribution of granite. The Laoshan Granite Belt, dominated by Laoshan granite, was formed by these intrusions. The entire Qingdao urban area is located on this type of granite, which provides favorable geological conditions for the construction of the subway.

**Figure 1:**
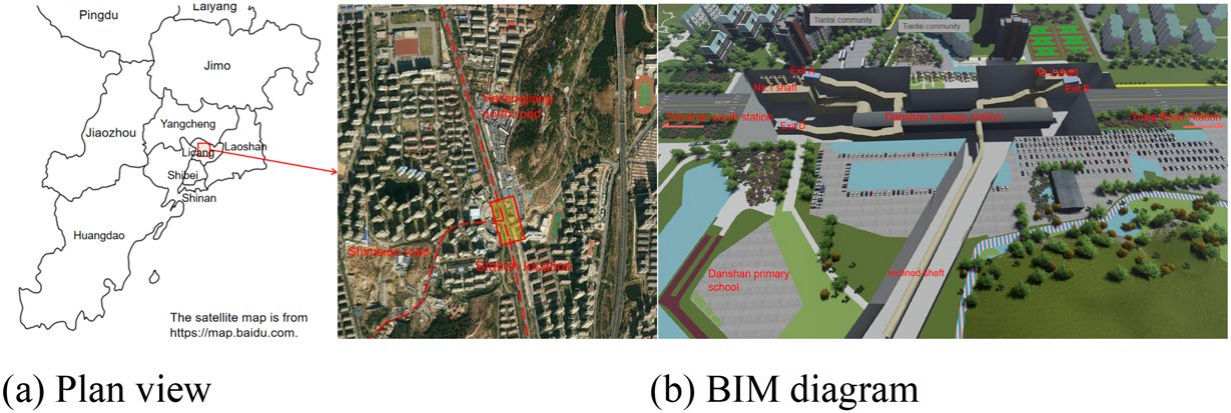
Plan of Danshan Station, Qingdao Metro.

**Figure 2:**
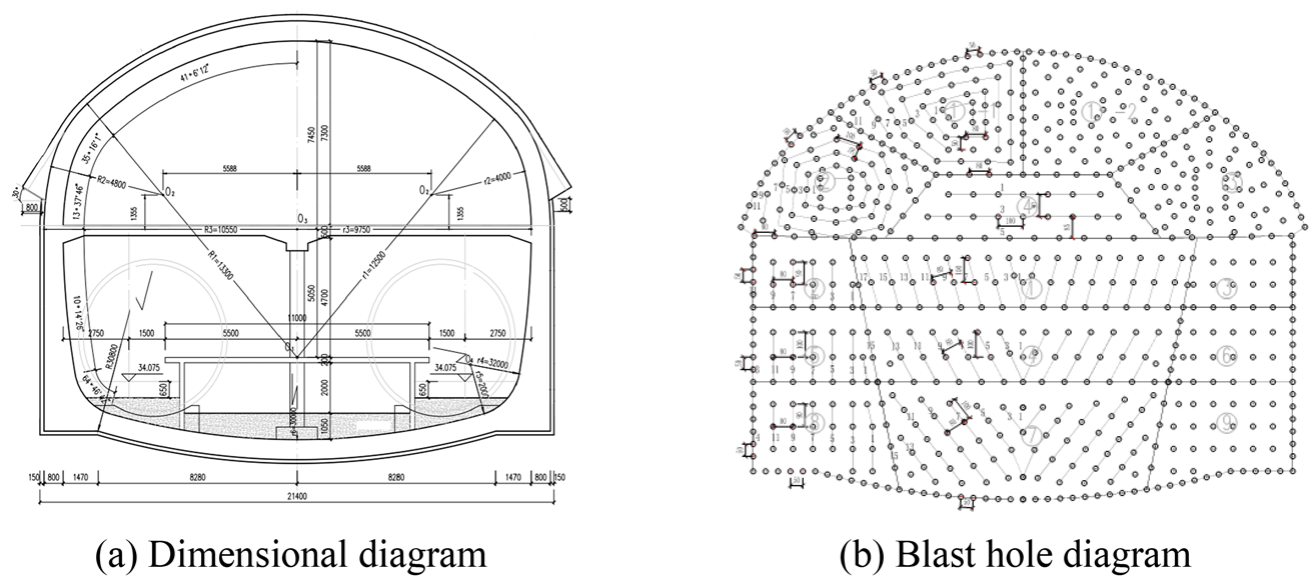
Layout of tunnel cross-section.

The Dan Mountain Station is located beneath Heilongjiang Road in the Chengyang District of Qingdao City. The rock strata at the site can be divided, from top to bottom, into strongly, moderately, and slightly weathered zones. The main part of the constructed tunnel is located in the moderately to slightly weathered granite belt of the Laoshan period, with an overlying rock thickness of 15.9–19.6 m. The Laoshan period granite formed during the late Yanshan period and is an A-type granite, which represents re-melted granite in the lower crust of the Early Cretaceous in eastern China. The subway station map and the tunnel cross-section diagram are shown in Figs. 1 and 2, respectively.

## JH-2 model theory and parameters

This paper investigates the dynamic response of tunnel lining structures under blasting conditions using the JH-2 model, developed by Holmquist and Johnson based on damage mechanics; The constitutive model takes into account the damage effect and can more accurately predict the failure behavior of the material under high strain rate and shock load conditions. This makes it have a wide range of applications in the field of engineering, so the JH-2 model is used to numerically simulate the surrounding rock. Recently, the JH-2 model has been extensively applied in various fields to predict material responses.

Determining the parameters of the JH-2 model requires conducting numerous experiments to evaluate the material strength, fracture toughness, strain rate sensitivity, and other parameters. The experimental data are then used to calibrate the model. The JH-2 model consists of three components: (1) the material strength model, which describes the strength response of the material under compressive loading; (2) the equation of state (EOS), which characterizes the volume response of the material under pressure; and (3) the material damage model, which describes the transition from an intact to a fractured state under plastic deformation conditions.

By employing the JH-2 model, a more accurate analysis of the dynamic response of the tunnel lining structures can be conducted under blasting conditions. This model provides designers with reliable and convincing results, thereby guiding the design and construction of tunnel blasting projects.

### Strength model

The normalized equivalent stress of “HEL” which is the normalized equivalent stress under the Hugoniot elastic limit (the critical value of maximum stress and strain a material can withstand when subjected to high-speed loading such as impact or explosion), is

1 $$\sigma^{*} = \sigma_{I}^{*} - D\left( {\sigma_{I}^{*} - \sigma_{F}^{*} } \right)$$

where ${\upsigma }_{{\text{I}}}^{*}$ denotes the normalized complete equivalent force, $\sigma_{F}^{*} { }$ denotes the normalized fracture stress, and D denotes the damage coefficient (0 ≤ *D* ≤ 1.0). The general form of the normalized equivalence force $(\sigma^{*}$, $\sigma_{I}^{*}$, $\sigma_{F}^{*} )$ is

2 $$\sigma^{*} = \sigma /\sigma_{HEL}$$

“$\sigma$” denotes the actual equivalent stress, which is calculated using the Von Mises stress formula.

3 $$\sigma^{*} = \frac{1}{2}\sqrt {\left[ {\left( {\sigma_{1} - \sigma_{2} } \right)^{2} + \left( {\sigma_{1} - \sigma_{3} } \right)^{2} + \left( {\sigma_{2} - \sigma_{3} } \right)^{2} } \right]}$$

The normalized complete strength is expressed as

4 $$\sigma_{F}^{*} = A\left( {P^{*} + T^{*} } \right)^{N} \times \left( {1 + C \times {\text{In}}\;\varepsilon^{*} } \right)$$

The normalized fracture strength is expressed as

5 $$\sigma_{F}^{*} = B\left( {P^{*} } \right)^{M} \times \left( {1 + C \times {\text{In}}\;\varepsilon^{*} } \right)$$

where, A, B, C, M, and N are constants. The normalized pressure is defined as P* = *P/P*_*HEL*_, where *P* is the actual pressure, and *P*_*HEL*_ is the pressure at the HEL. The normalized maximum tensile hydrostatic pressure is T* = *T/P*_*HEL*_, where *T* is the maximum tensile hydrostatic pressure that the material can withstand. The normalized strain rate is $\varepsilon^{*}$ = $\varepsilon$/$\varepsilon_{0}$, where $\varepsilon^{*}$ is the actual strain rate of the material, and $\varepsilon_{0}$ = 1s^−1^ is the reference strain rate of the material.

### Equation of state (EOS)

When a material undergoes compression, the EOS is used to define the relationship between the hydrostatic pressure *P* and volumetric strain μ of the material. For a complete material, the EOS is defined as

6 $$P = K_{1} \mu + K_{2} \mu^{2} + K_{3} \mu^{3}$$

where $\mu = \rho /\rho_{0} - 1$, *K*_1_ is the bulk modulus, and *K*_2_ and *K*_3_ are pressure constants. The volumetric strain is, where $\rho$ is the current density and $\rho_{0}$ is the initial density. When the rock is subjected to tensile stress, μ becomes negative for tensile pressures, in which case ${\text{P}} = {\text{K}}_{1}\upmu$. When the rock strength reaches its maximum value and the degree of material damage increases, the state equation adds the incremental pressure $\Delta P$.

7 $$P = K_{1} \mu + K_{2} \mu^{2} + K_{3} \mu^{3} + \Delta P$$

### Damage model

The amount of plastic strain required for a material to transition from an intact to a fractured state depends on the pressure. The equivalent plastic strain at which the model fractures is obtained using the following equation:

8 $$\varepsilon_{P}^{F} = D_{1} \left( {P^{*} + T^{*} } \right)^{{D_{2} }}$$

$D_{1} ,D_{2} ,P^{*} ,T^{*}$ have been defined earlier. When a material undergoes plastic deformation, damage accumulates within it, and its value can be calculated using the following equation:

9 $$D = \sum \frac{{\Delta \varepsilon_{P} }}{{\varepsilon_{P}^{F} }}$$

where 1 represents the increment in the equivalent plastic strain within a computational cycle. When the equivalent stress is relatively low, the material remains in the elastic region without undergoing plastic deformation, thereby maintaining its integrity ($D =$ 0). When the equivalent stress is high, the material undergoes permanent deformation, resulting in an overall increase in the equivalent plastic strain and a decrease in the material strength (0 < *D* < 1.0). When the equivalent stress becomes excessively high, the material strength decreases to the fracture strength and the equivalent plastic strain becomes equal to the fracture strain, resulting in complete material damage ($D =$ 1).

### Parameter determination

**Figure 3:**
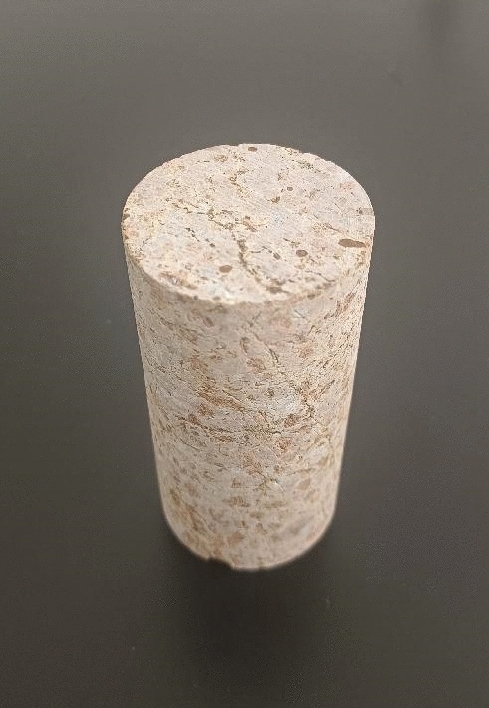
Sample of mildly weathered granite.

**Figure 4:**
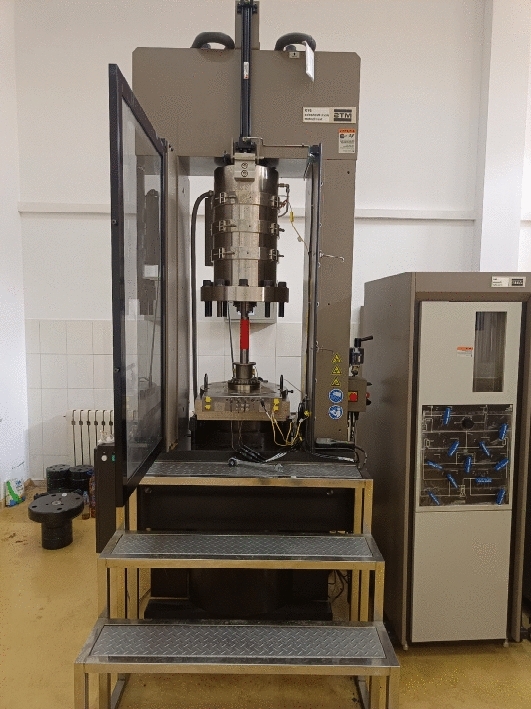
MTS rock compression machine.

Representative rock samples from slightly weathered Qingdao rock formations were collected through on-site excavation, processed, and cut into cylinders. The samples were cylindrical with a diameter of 50 mm and height of 100 mm. The experimental samples and instruments are shown in Figs. 3 and 4, respectively.

Three natural samples were subjected to uniaxial compression tests and the average value was used as the strength parameter for the granite. The results of the uniaxial compression tests on the rock are listed in Table 1 below.

**Table 1:** Strength parameters of microweathered granite.

Test no	Uniaxial compressive strength(MPa)	Average uniaxial compressive strength(MPa)	E/(GPa)	Average value of E/(GPa)
1	118.87	113.51	20.04	20.85
2	109.16		17.51	
3	112.50		25.01	

The shear modulus G was calculated using the formula $G = E/\left( {1 = 2 \upsilon } \right)$, and the bulk modulus K_1_ was calculated using the formula $K_{1} = E\left[ {3 \times \left( {1 - 2} \right)\upsilon } \right]$. The parameters of the physical rock samples obtained through calculations and comparisons with other researchers' results are shown in Table 2.

**Table 2:** Basic parameters of surrounding rock.

$\rho$/(kg/m3)	E/(GPa)	$\upsilon$	G/(GPa)	T/MPa	K	C/MPa	Vp/(km/s)	Vs/(km/s)	Rc/(MPa)
2540	20.85	0.26	8.14	4.8	30.02	1.41	4.70	3.71	113.51

In the JH-2 constitutive model, the HEL is an important concept that permeates the entire calculation process. Yuan^23^ estimated the HEL of Westerly granite to be 3.2–3.5 GPa through plate impact tests conducted in 2013. Wu^24^ also adopted values within this range and verified them through numerical simulations. Therefore, this study used HEL = 3.2 GPa as the experimental parameter in the simulation.

10 $${\text{P}}_{{{\text{HEL}}}} = \frac{{V_{p}^{2} }}{{V_{p}^{2} }} \times \frac{{\sigma_{HEL} }}{2}$$

11 $$\sigma_{HEL} = \frac{3}{2}\left( {HEL - P_{HEL} } \right)$$

12 $$P_{HEL} = K_{1} \mu_{HEL} + K_{2} \mu_{HEL} + K_{3} \mu_{HEL}$$

13 $$HEL = {\text{K}}_{1} {\upmu }_{{{\text{HEL}}}} + {\text{K}}_{2} {\upmu }_{{{\text{HEL}}}} + {\text{K}}_{3} {\upmu }_{{{\text{HEL}}}} + \frac{4}{3}G\left( {\frac{{\mu_{HEL} }}{{1 + \mu_{HEL} }}} \right)$$

By solving the above equations simultaneously, we can obtain

$${\upsigma }_{{{\text{HEL}}}} = {3}.0{1},\quad {\text{P}}_{{{\text{HEL}}}} = {2}.{49},\quad {\upmu }_{{{\text{HEL}}}} = 0.0{689}$$

In the absence of plate impact tests, Wang^21^ proposed a method for solving the constants *K*_2_ and *K*_3_ based on the principles of conservation of mass and momentum. By combining the shock wave velocity and particle velocity curves with the conservation equations and Hugoniot relationship, we obtain

14 $${\text{P}} = \frac{{{\rho C}^{2} {\upmu }\left( {1 + {\upmu }} \right)}}{{\left[ {1 - \left( {{\text{s}} - 1} \right){\upmu }} \right]^{2} }}$$

The body wave velocity, *C*, is given by

15 $$C = \sqrt {V_{P}^{2} - \frac{4}{3}V_{S}^{2} } = 1.96\;{\text{km}}/{\text{s}}$$

Substituting $\rho ,P_{HEL} ,\mu_{HEL} ,C$ into the above equation, we obtain $S = 7.05$. Therefore, Eq. (14) becomes

16 $$P = \frac{{2.54 \times 1.96^{2} \mu \left( {1 + \mu } \right)}}{{\left[ {1 - \left( {7.05 - 1} \right)\mu } \right]^{2} }}$$

**Figure 5:**
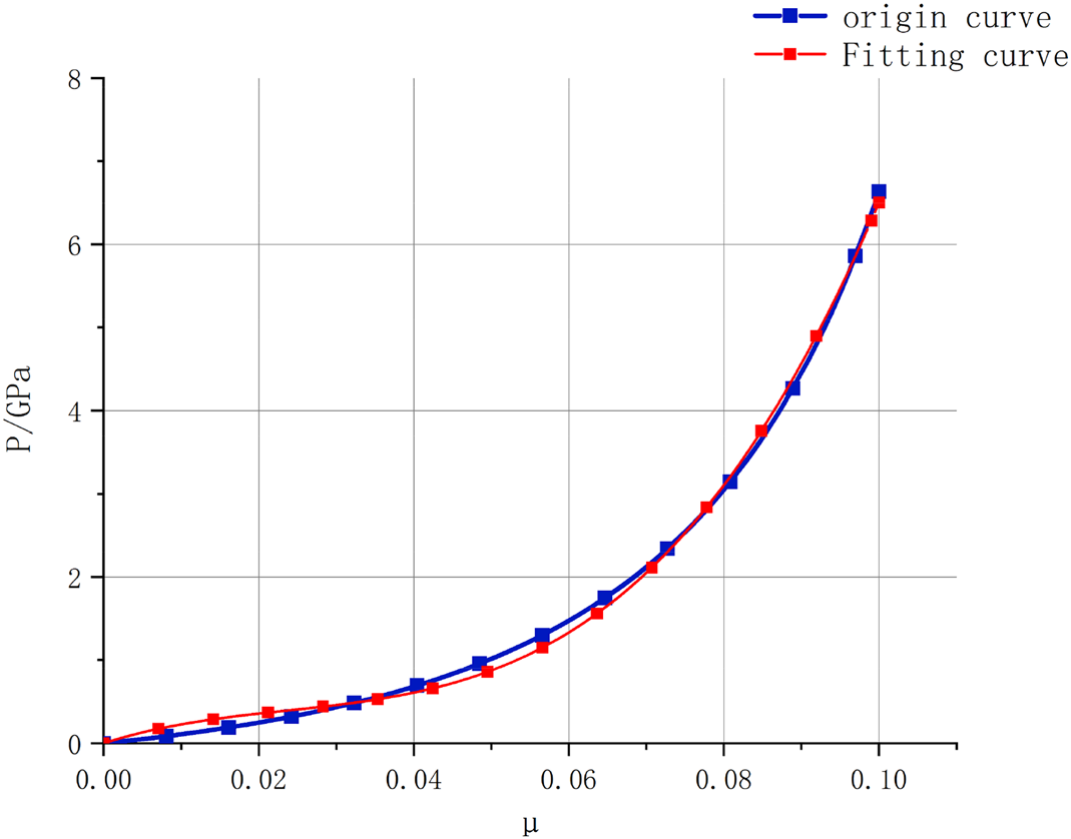
P-μ Fitted curve.

The P–μ curve obtained using Eq. (16) is shown in Fig. 5 below:

The curve was fitted using Eq. (6), and the fitting results were as follows: *K*_1_ = 30.02 GPa, *K*_2_ = − 857.20 GPa, *K*_3_ = 1132.50 GPa.

### Strength determination

Conducting high-confining-pressure triaxial tests on rock materials is challenging. Therefore, in this study, the Hoek–Brown criteria^25^ were used to predict the stress state of the rocks under different confining pressures. The Hoek–Brown criteria can fit a complete strength model and were originally developed to estimate the strength of hard rock masses.

17 $$\sigma_{1}{\prime} = \sigma_{3}{\prime} + \sigma_{ci} \left( {m_{i} \frac{{\sigma_{3}{\prime} }}{{\sigma_{ci} }} + s} \right)^{\frac{1}{2}}$$

where $\sigma_{1}{\prime}$ and $\sigma_{3}{\prime}$ are the maximum and minimum effective stresses at failure, $\sigma_{3}{\prime}$ is the uniaxial compressive strength of intact rock, and $s = 1$ for intact rock. $m_{i}$ represents the Hoek–Brown constant for the rock mass, with a value of $m = 32$ for granite, based on Reference^26^. Substituting these values into the formula, we obtain

18 $$\sigma_{1} = \sigma_{3} + 113.51\left( {32\frac{{\sigma_{3} }}{113.51} + 1} \right)^{\frac{1}{2}}$$

According to the fitting formula, assuming $\sigma_{2} = \sigma_{3}$ and applying a range of 0–1200 MPa, we obtain $\sigma_{1}$. The normalized stress $\sigma_{1}$ can be calculated using Eqs. (2) and (3), and the normalized hydrostatic pressure P* can be calculated using the following formula:

19 $$P = \frac{{\sigma_{1} + \sigma_{2} + \sigma_{3} }}{3}$$

20 $$P^{*} = P/P_{HEL}$$

The normalized equivalent stress and normalized hydrostatic pressure values for granite in the Qingdao region under different confining pressures are presented in Table 3 below.

**Table 3:** Normalized equieffect forces and normalized hydrostatic pressure calculations.

${\sigma }_{2}={\sigma }_{3}$/(MPa)	${\sigma }_{1}$/(MPa)	P/(MPa)	$\sigma$/(MPa)	${P}^{*}=P/{P}_{HEL}$	${\sigma }^{*}=\left(\sigma /{\sigma }_{HEL}\right)$
-3.50	9.44	0.81	12.94	-0.00093	0.0012
0.00	113.50	37.83	113.50	0.02	0.04
25.00	347.03	132.34	322.03	0.05	0.11
50.00	491.05	197.02	441.05	0.08	0.15
100.00	713.32	304.44	613.32	0.12	0.20
150.00	896.86	398.95	746.86	0.16	0.25
200.00	1059.91	486.64	859.91	0.19	0.29
300.00	1350.11	650.04	1050.11	0.26	0.35
400.00	1610.78	803.59	1210.78	0.32	0.40
500.00	1852.51	950.84	1352.51	0.38	0.45
600.00	2080.73	1093.58	1480.73	0.44	0.49
700.00	2298.70	1232.90	1598.70	0.49	0.53
800.00	2508.54	1369.51	1708.54	0.55	0.57
900.00	2711.74	1503.91	1811.74	0.60	0.60
1000.00	2909.37	1636.46	1909.37	0.65	0.64
1100.00	3102.24	1767.41	2002.24	0.71	0.67
1200.00	3290.99	1897.00	2090.99	0.76	0.70

**Figure 6:**
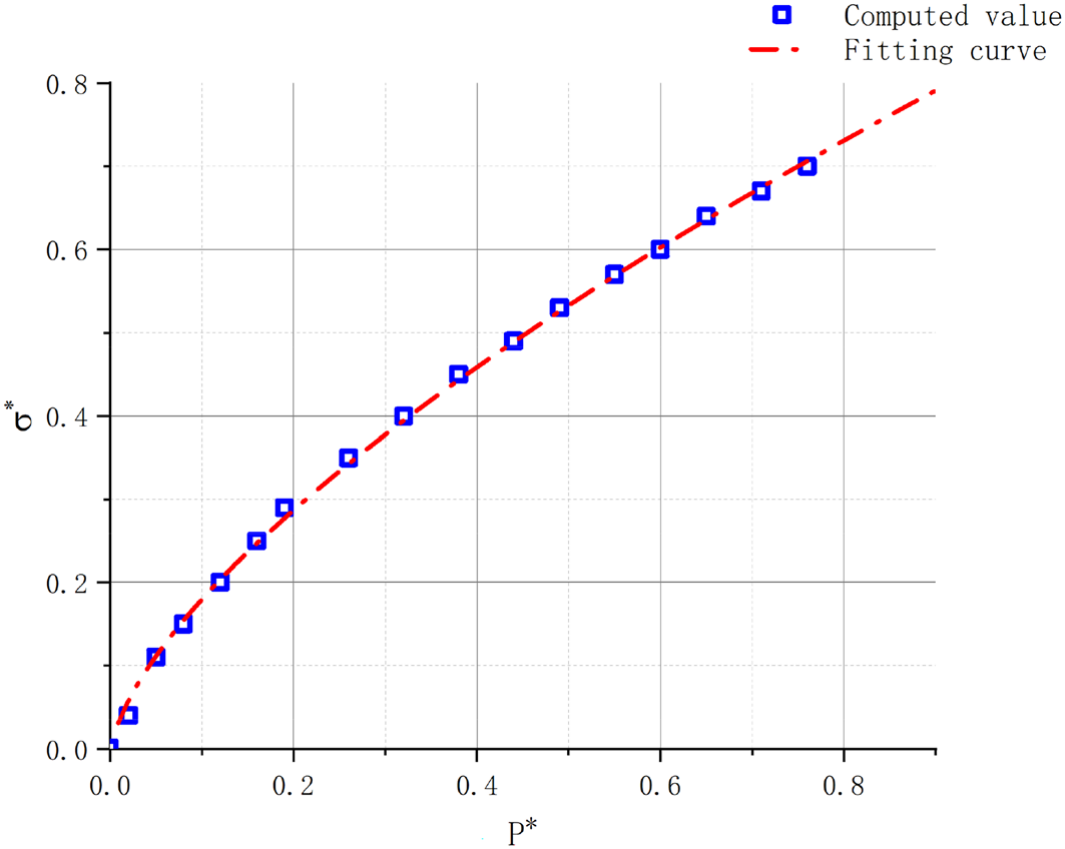
P*-fitted curve.

The curve of the fitted relationship between the normalized hydrostatic pressure P* and normalized stress 2 for granite can be obtained by calculating the parameters. The curve of fitting relationship is shown in Fig. 6 below.

Because of the low strain rate in the triaxial compression experiments, the dynamic correction factor $\left( {1 + C \times In\varepsilon^{*} } \right)$ was not considered in this case. Through fitting the curve, the obtained values were *A* = 0.85, *N* = 0.67, and T* = 0.00192 $\left( {T = T^{*} \times P_{HEL} = 48MPa} \right)$. According to current academic consensus, the rock failure strength is one-third of the intact strength; therefore, *B* = 0.28. The relationship between constants *M* and *N* is commonly taken as *M* = *N* = 0.67. Several researchers have obtained similar results for the constant C in granite by fitting the P–σ relationship using dynamic compression test data. Therefore, based on previous research, this study adopted a value of *C* = 0.005. M. Banadaki^19^ numerically adjusted the damage parameters of granite materials and determined *D*1 = 0.005 and *D*2 = 0.7 as the most suitable values.

The parameters of the JH-2 igneous granite in the Qingdao region are listed in Table 4 below.

**Table 4:** Granite JH-2 parameters.

$\rho$/(kg/m3)	*G*/GPa	*A*	*B*	*N*	*C*	*M*	$\dot{{\varepsilon }_{0}}$
2540	8.4	0.85	0.28	0.67	0.005	0.67	1
*T*/(MPa)	*D*1	*D*2	*HEL*/GPa	*K*_1_	*K*_2_	*K*_3_	*P*_*HEL*_/GPa
48	0.005	0.7	3.20	30.02	-857.20	1132.50	2.59

Owing to the relatively fixed strength properties of concrete, this study adopted the JH-2 parameters for concrete from a previous study^27^ for the simulation. The specific values are listed in Table 5 below.

**Table 5:** JH-2 parameters of C30 concrete.

$\rho$/(kg/m3)	*G*/GPa	*A*	*B*	*N*	*C*	*M*	$\dot{{\varepsilon }_{0}}$
2440	14.9	0.79	1.60	0	0.007	0.61	1
*T*/(MPa)	*D*1	*D*2	*HEL*/GPa	*K*_1_	*K*_2_	*K*_3_	*P*_*HEL*_/GPa
3.5	0.04	1.0	1.48	85.00	-171.00	208.00	48

## Numerical calculation model and reliability analysis

### Numerical calculation model and analysis methods

**Figure 7:**
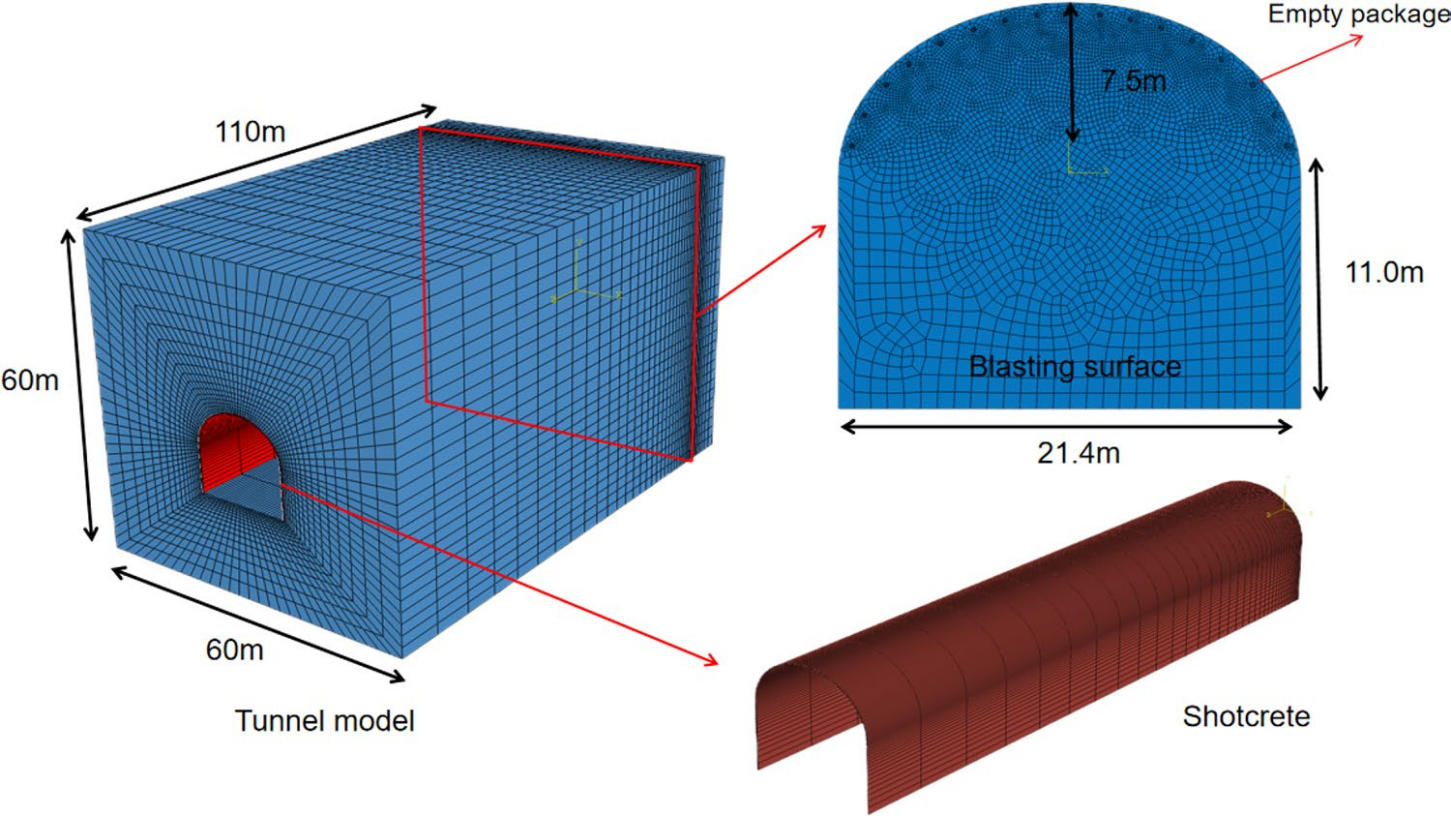
Three-dimensional model of tunnel surrounding rock-initial lining concrete.

Using the finite-element program Abaqus, a three-dimensional geomechanical model of a large-section tunnel under blasting construction was established. In the numerical simulation, the tunnel was assumed to be excavated in a homogeneous isotropic granite medium. The models of the surrounding rock and lining structure are shown in the figure below. Shotcrete is often used as the initial support of tunnels in blasting engineering, so the key lining structure in numerical simulation refers to shotcrete. The size of the surrounding rock model was 60 m × 60 m × 110 m, with a tunnel width of 21.4 m and a height of 18.5 m. The excavation progress of the tunnel was 100 m. Because the outer perimeter blast hole is closest to the surrounding rock and lining structure of the tunnel, this area is most prone to engineering problems in the actual project^28^, and considering the computer computing power, only the outer perimeter blast hole that has the greatest impact on the lining structure is considered in the simulation. The blast hole was a cylindrical space with a size of 0.06 m × 1.2 m, with a slightly smaller diameter than the blast hole of emulsion explosive, and its distribution was located in the region of the tunnel length from 100 to 101.2 m. The outer surface of the lining structure was tightly attached to the surrounding rock with a length of 100 m and thickness of 0.2 m. The end of the lining structure was extended to the blasting face of the tunnel to better explore the safe distance of the concrete during blasting. A general contact friction coefficient of 0.6 was used between the surrounding rock and concrete, and the element type for the surrounding rock and concrete was a Lagrange element (C3D3R). To simplify the calculation, we simplified the mesh appropriately, and the overall distribution was denser the closer it was to the blast source. The rock-support model diagram is shown in Fig. 7.

**Figure 8:**
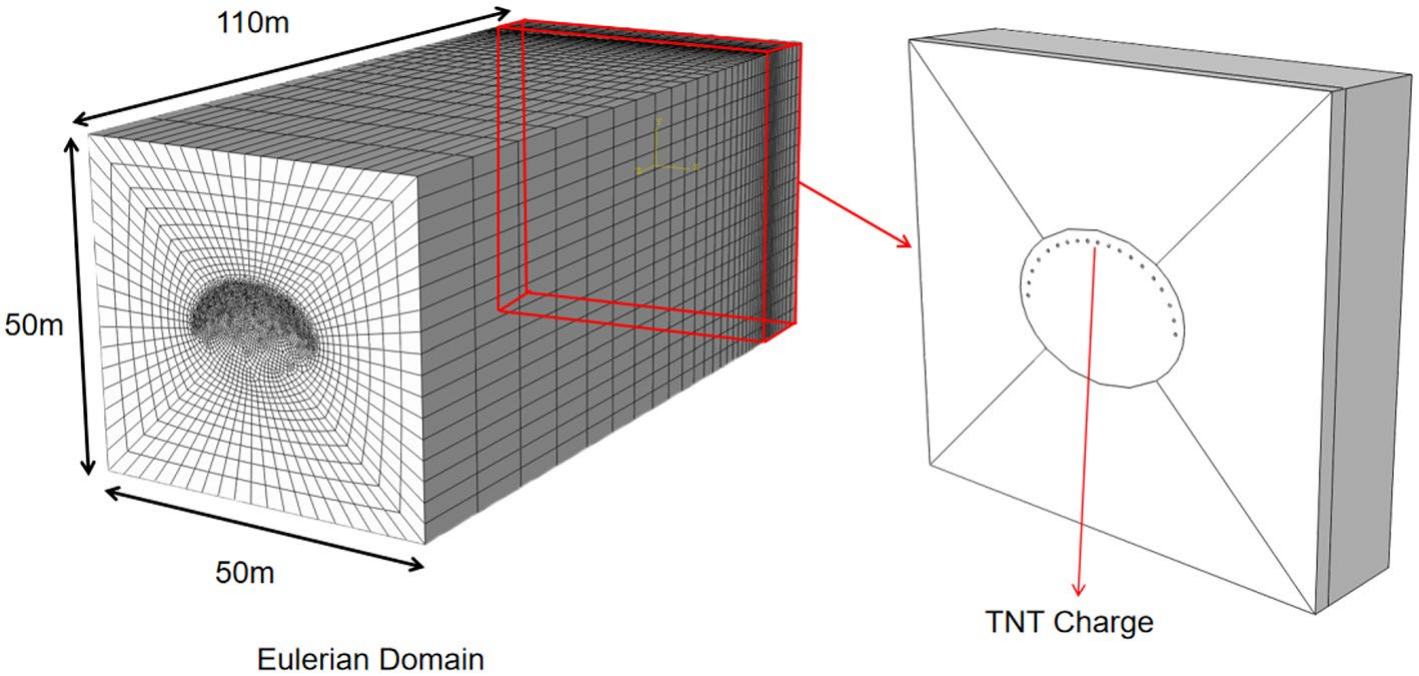
Lagrangian element and Eulerian model.

The Eulerian component was divided into Lagrangian elements and Eulerian domains using the coupled Eulerian–Lagrangian (CEL) method. The Lagrangian domain was set as the TNT, whereas the Eulerian domain was set as the air material, enabling the transmission of shockwaves generated by the explosive within the tunnel space. As shown in the figure below, the dimensions of the Eulerian domain model were 50 m × 50 m × 110 m, and the TNT had the shape of a cylindrical body with dimensions of 0.06 m × 0.6 m. It was positioned at the rear end of the model, specifically at 10.0–10.6 m. The mass of the charge is 1.3 kg, which corresponds to 15 m^3^ of surrounding rock around the blast hole. The TNT model was precisely positioned at the center of the blast hole after assembly, and the explosive models were arranged according to actual engineering practices and simultaneously detonated at the start of the simulation. The TNT and air were modeled using eight-node reduced integration Eulerian elements (EC3D8R). Nonreflecting boundary conditions were applied to the boundaries of the Eulerian domain to eliminate reflections caused by the interaction of shock waves with the boundaries. By simulating the interaction between air and explosives using the CEL method and coupling it with the JH-2 model for rock concrete, the stress waves and combined effects of the explosive gases generated by detonation can be effectively simulated. When an explosive is detonated in a rock borehole, the surrounding rock medium experiences an instantaneous impact stress that exceeds its compressive strength, leading to rock fragmentation under the action of expanding gases. Stress waves are transmitted through the air in the tunnel, and this process can be visually displayed using the damage nephogram output by Abaqus. The Lagrange element and Euler domain models are shown in Fig. 8.

The Euler body consists of both the JWL explosive and air components. The parameters for the emulsion explosive and air are listed in Tables 6 and 7, respectively. The detonation delay and initial XY position of the JWL explosive were set to 0. Owing to the grid resolution, the TNT had a cylindrical shape with dimensions of 0.06 m × 0.6 m, which closely matches the size of emulsion explosives commonly used in actual engineering for tunnel blasting. The calculated mass of a single explosive was 1.38 kg.

**Table 6:** Emulsion explosive parameters.

$\rho$/(kg/m^3^)	Detonation wave speed (m/s)	*A*/(MPa)	*B* /(MPa)	$\omega$	*R*_1_	*R*_2_	Detonation energy density /(J/kg)
1630	6930	557,600	5350	0.35	6.01	1.07	6,060,000

**Table 7:** Air parameters.

$\rho$/(kg/m^3^)	Gas constant	Environmental pressure/(Pa)	Specific heat capacity	Dynamic viscosity
1.01	6930	10,000	717.7	8.25 × 10^–5^

### Reliability analysis

For the JH-2 model, many studies have proved the accuracy of the PPV data in blasting simulation^21^, but there is still a gap in the reliability of the stress field analysis of the surrounding rock and lining structure, and it is difficult to obtain the stress interaction results between the tunnel lining structure and the surrounding rock mass due to on-site monitoring. Therefore, in order to verify the reliability of the JH-2 model in the propagation of stress waves, the accuracy of the numerical simulation of the stress field is verified by calculating the analytical solution by the PPV theoretical formula. According to several scholars^6,29,30^, the dynamic stress can be calculated using the PPV. The calculation formula for dynamic stress σ of the material is expressed as follows:

21 $$\sigma = K_{n} \times PPV \sigma = K_{n} \cdot PPV$$

22 $$K_{n} = \rho \times V_{p}$$

23 $$V_{p} = \sqrt {\frac{E}{\rho }}$$

By combining the above equations, we can obtain

24 $$\sigma = \sqrt {E\rho } \times PPV$$

**Figure 9:**
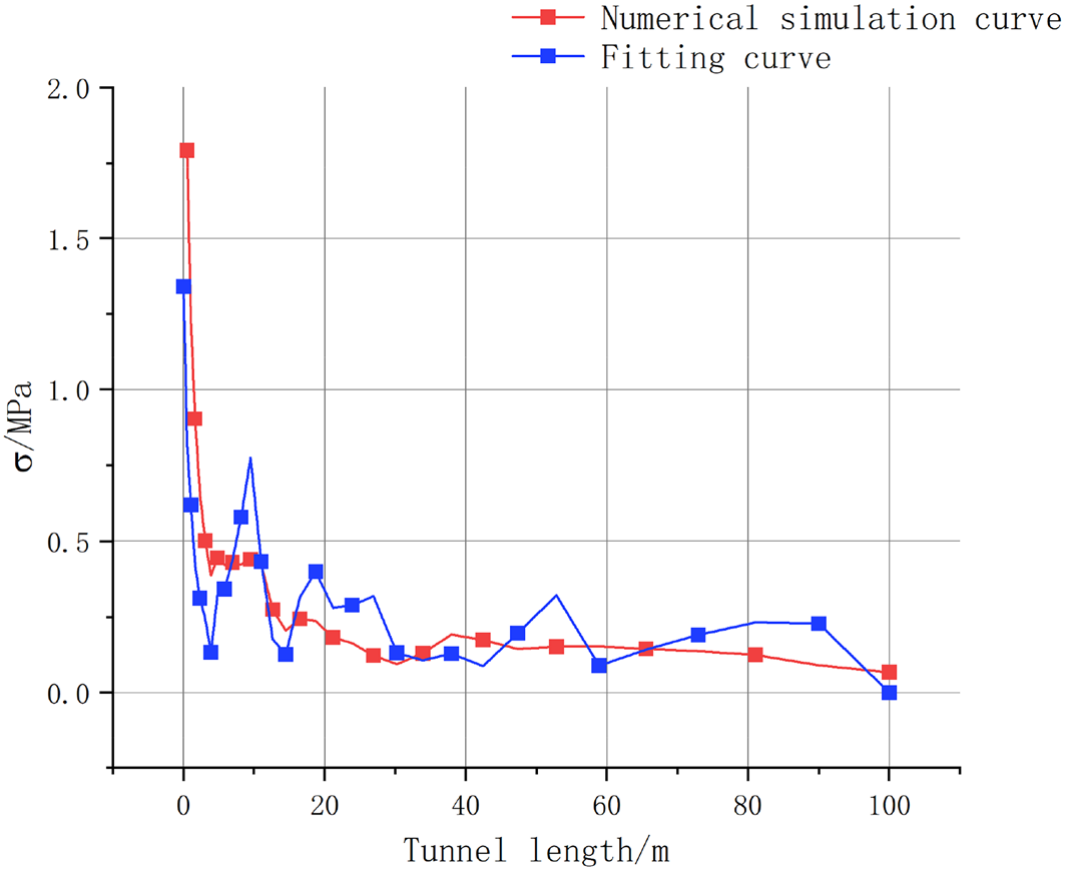
Fitted curve of surrounding rock stress.

For the selected arch path, the vibration velocity data of the concrete and surrounding rock after 100 ms of blasting were selected. After the calculation, the data were fitted to the simulated stress curve, as shown in the Fig. 9 below. The fitted results indicate that the simulated effective stress and stress variation calculated using the vibration velocity were essentially the same, with the error controlled within 15%. Considering the fluctuation in the vibration velocity in the numerical simulations, the analytical solution derived from the formula is compared with the stress of the lining structure, which proves the accuracy and reliability of the correlation between PPV and stress of the JH-2 constitutive model.

## Analysis of dynamic response characteristics of the lining structure

### Stress wave transmission law

**Figure 10:**
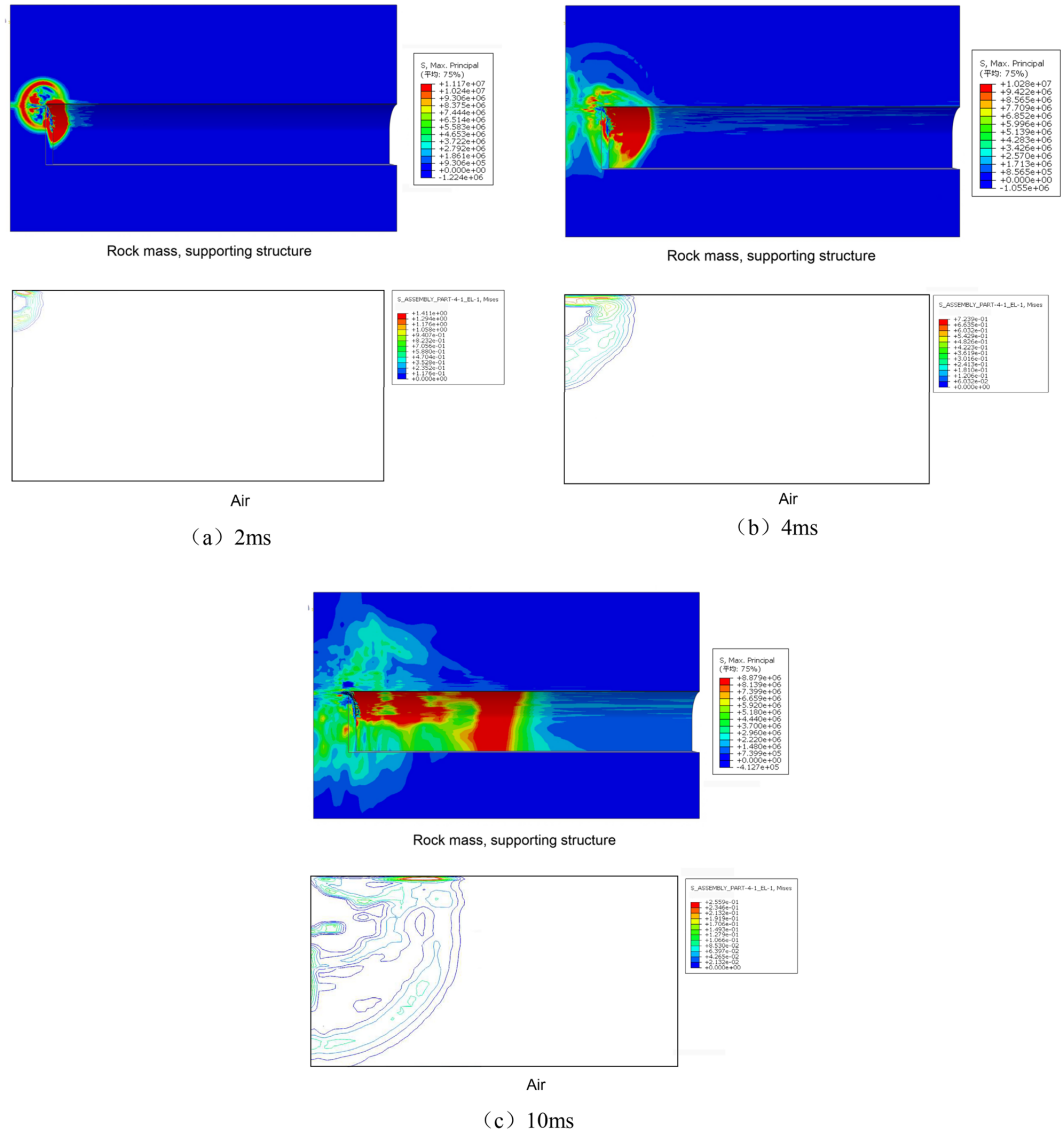
Stress nephogram during blasting process.

The stress isocontour map of the rock–mass lining structure during the blasting process is shown in the Fig. 10 below. After blasting, a large amount of high-temperature and high-pressure gas was generated by the emulsion explosive. In the local high-pressure area inside the blast hole, a shock wave was formed at the blasting source, and the shock load first acted on the borehole wall. The propagation speed of the shock wave was much higher than the development of cracks around the borehole. From the figure, the following observations can be made. (1) The propagation speeds of stress waves in different media are different, with the propagation speed in air being significantly slower than that in solid media. The transmission of the stress waves in the lining structure is significantly faster than that in the tunnel rock mass. (2) Within 2 ms of blasting, the stress waves in the concrete and rock masses were of the same magnitude. However, after 4 ms, the stress in the support structure was greater than that in the surrounding rock mass, indicating that the attenuation rate of the stress waves in the rock mass was faster. (3) Advanced diffusion of stress waves was observed at the interface between the concrete and surrounding rock mass, which is speculated to be due to the different strengths of the media models. (4) Within 10 ms of blasting, the stress in the air rapidly attenuated. The peak stress in air was concentrated above the blasting source near the upper wall of the tunnel.

### Stress at the bonding surface

In the support of the lining structure, the bonding surface between the lining and surrounding rock mass is the weakest owing to the mechanical performance differences between them. This location is the most susceptible to damage, and to ensure that the concrete lining remains intact, shear or tensile failure should be prevented at the bonding surface between the concrete and the surrounding rock mass^9^.

1. Attenuation law

The propagation process of stress waves is extremely complex. When different media interfaces or spatial structures are encountered, reflection and refraction occur, changing the propagation direction and energy distribution of the waves, which can cause tensile or shear failure in the medium. According to a study by Ming et al.^7^, the incident angle is an important factor affecting the safety of the PPV. The difference in the stress wave propagation speed on both sides of the bonding surface can lead to increased local stress, thereby increasing the damage and risk of failure in the medium. Therefore, the impact of the stress concentration caused by the different velocities on both sides of the medium is greater at the bonding surface. According to Eq. (15), the P-wave velocity of the granite used in the simulation was 1.96 km/s, and the P-wave velocity of the C30 concrete was calculated as 3.50 km/s based on empirical formulas. The P-wave velocity is an important parameter for calculating the bulk modulus K; a higher K results in higher force conduction and sound wave propagation speed. Therefore, the faster stress wave transmission in the lining structure is caused by its higher elastic and bulk moduli.

**Figure 11:**
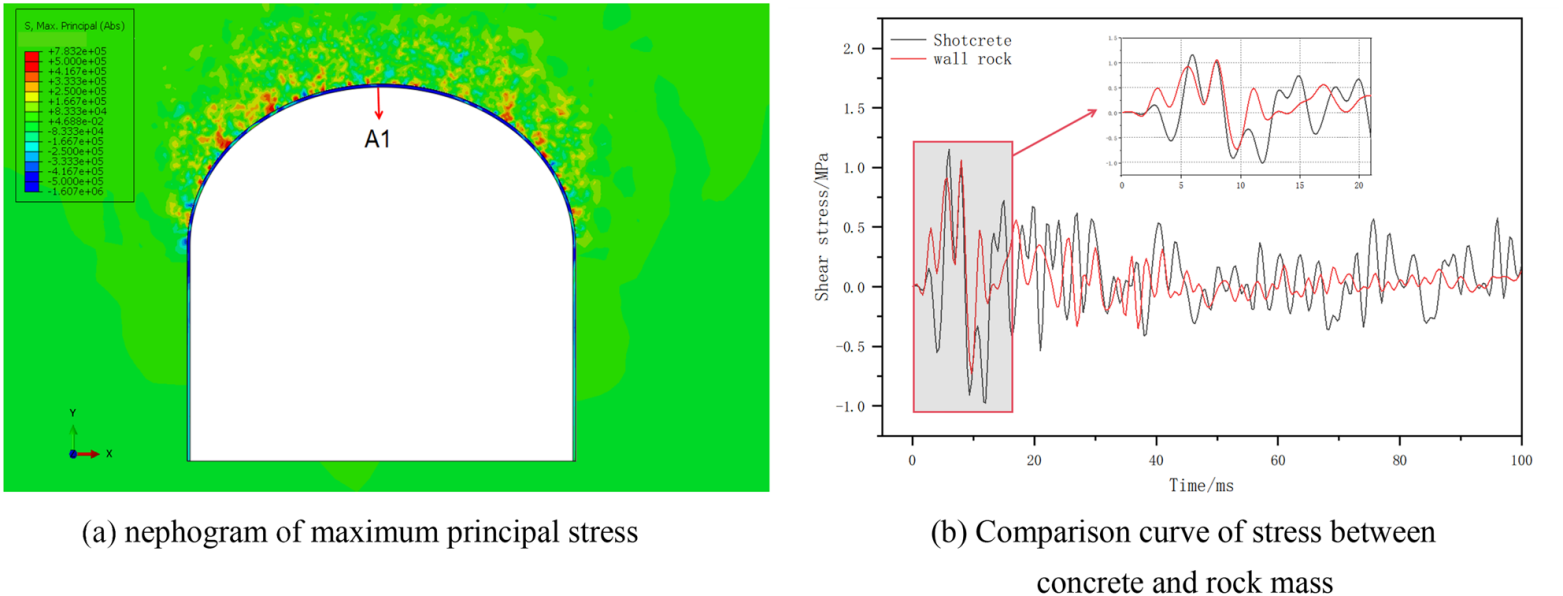
Schematic diagram of pre-stressing.

To investigate the transmission law of the advanced stress waves at the bonding surface, we selected the XY section at a distance of 20 m from the blasting source in the tunnel at 3 ms for observation. By comparing the data, we observed that the maximum advanced stress occurred in this section. A nephogram of the maximum principal stress is presented in Fig. 11a. Point A1, located at the bonding surface between the concrete and rock mass in the section above, was selected. A comparison of the time-history curves of the principal stresses in the concrete and surrounding rock on both sides of A1 is shown in Fig. 11b.

Based on the maximum principal stress nephogram in Abaqus, a complex stress distribution occurred at the intersection of the surrounding rock mass and the concrete in the XY section. The type of stress on the XY section was shear stress, ranging from − 1.61 to 0.78 MPa. A positive value indicates that the concrete and the rock mass have a tendency to slide against each other, while a negative value indicates that the concrete and rock mass are squeezing each other. Figure (b) shows that within 0–12 ms after blasting, the variations in shear stress in the surrounding rock and lining structure were similar, except for the stress amplitude. The propagation laws of the stress waves were identical. After 12 ms, the frequencies of the two were observed to be different, and the attenuation of the stress peak in the lining structure was more apparent. At 8 ms, the stress wave reached the interface area, with a peak stress of 1.06 MPa, and it attenuated to 0.07 MPa at 100 ms, with an amplitude of 93.4%. The surrounding rock stress decreased from 1.16 MPa at 6 ms to 0.19 MPa at 100 ms, with an amplitude of 83.6%. At 11 ms, the stress difference between the concrete and the surrounding rock is the largest, with a magnitude of 0.91 MPa. According to^9^, the safe stress range for a bonding surface varies depending on surrounding rock conditions. The safety stress can be calculated using Eq. (25).

25 $$\sigma_{b} = 2.345\sigma_{24b} e^{{ - 0.858t^{ - 0.97} }}$$

For different rock grades (II, III, IV, and V), the values are not less than 0.8, 0.5, 0.42, and 0.31 MPa, respectively^9^. Here, t represents the age of the lining structure in days. Based on the calculations, the bonding strength between the slightly weathered granite and concrete at the bonding surface was 1.12 MPa, which was greater than the maximum stress difference at the bonding surface. This proves that under the given blasting conditions, the stress concentration caused by the different propagation speeds of the stress waves between different media did not damage the concrete–rock bonding surface.

2. Comparative analysis of different lining materials

**Figure 12:**
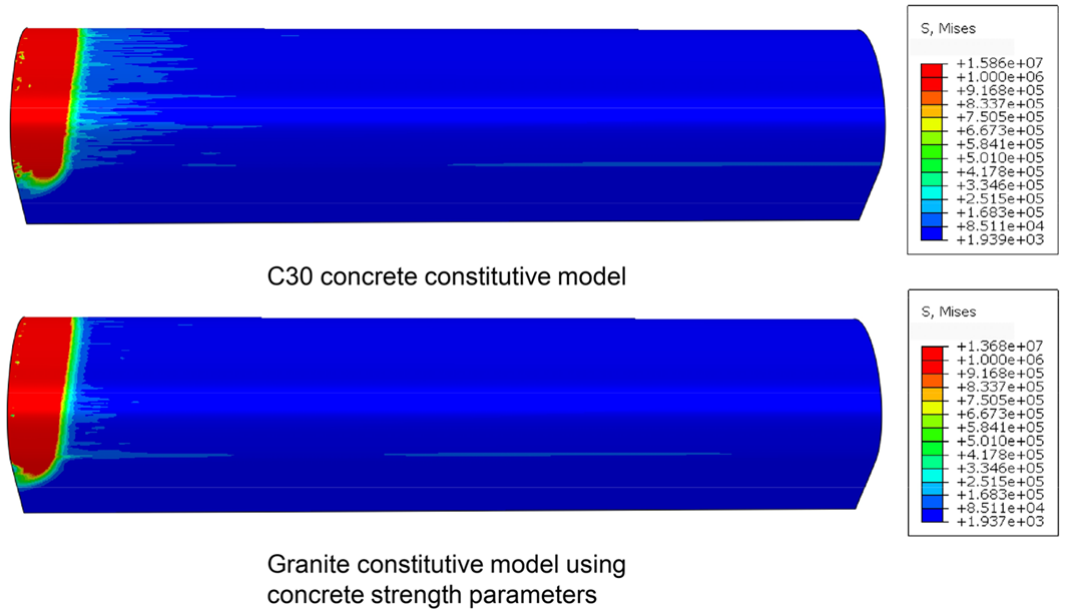
Stress nephogram of lining structure.

**Figure 13:**
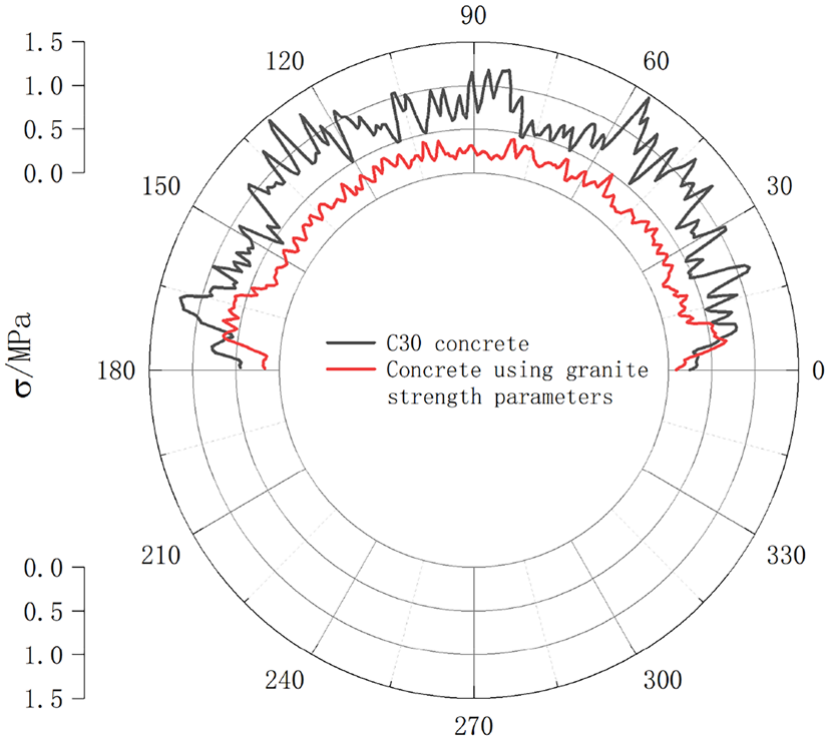
XY section shear stress nephogram.

To verify the stress differences caused by different medium velocities, in the simulation, we modified the JH-2 parameters of the lining structure material, except for the shear modulus, to those of the weathered granite material for the surrounding rock. The other parameters remained unchanged. The stress nephogram of the lining structure is shown in Fig. 12. The peak stress in both cases decreased from 15.86 to 13.68 MPa with a reduction of 13.75%. The nephogram shows that when using C30 concrete for the support structure, a clear and rapid wave load occurred with stress diffusion at the interface of the stress wave. However, when using the modified parameter material, the stress-wave interface had a clear boundary, and no noticeable stress-wave diffusion occurred beyond the stress of the surrounding rock mass. A circular stress curve of the lining structure was constructed at a section 15 m into the tunnel to quantitatively analyze this stress wave diffusion, as shown in Fig. 13.

Figure 13 shows that there were significant differences in the circumferential stress distributions in the two cases. When using C30 concrete material, a larger stress diffusion range was observed, with fluctuations ranging from 0.44 to 1.48 MPa, with an average value of 0.80 MPa. The stress peak occurred in the ranges of 30°–60° and 120°–150° on both sides of the crown. Therefore, in practical engineering, the bonding effect between the concrete and surrounding rock should be considered within this range to prevent concrete spalling and separation. In contrast, when using the modified parameter concrete material, the diffusion stress range was 0.18–0.70 MPa, with an average value of 0.30 MPa. The distribution of the stress peaks was in the ranges of 0°–15° and 160°–175°. Compared with the C30 concrete material, the support structure with the modified parameter material exhibited a smaller stress concentration, with a reduction of 52.7% in the peak stress. This reduction was greater than that of the stress waves generated by blasting. Therefore, without considering the strength of the support structure, lining materials with strength parameters similar to those of the surrounding rock can effectively reduce damage to the concrete–rock bonding surface.

### Prediction of the peak particle velocity (PPV) for blasting in lining structure

1. Prediction of safe distance for the lining structure

The PPV has been widely studied and applied in blasting engineering. Many studies have used it to describe the vibration effects of explosions and assess the damage to structures during tunnel blasting. Various researchers have proposed various fitting formulas for the PPV. Because of the complexity of the blasting conditions in this simulation, the Sadovsky formula, which considers the design parameters of an explosion, was adopted. This formula is widely used in China to examine PPV propagation and attenuation.

**Figure 14:**
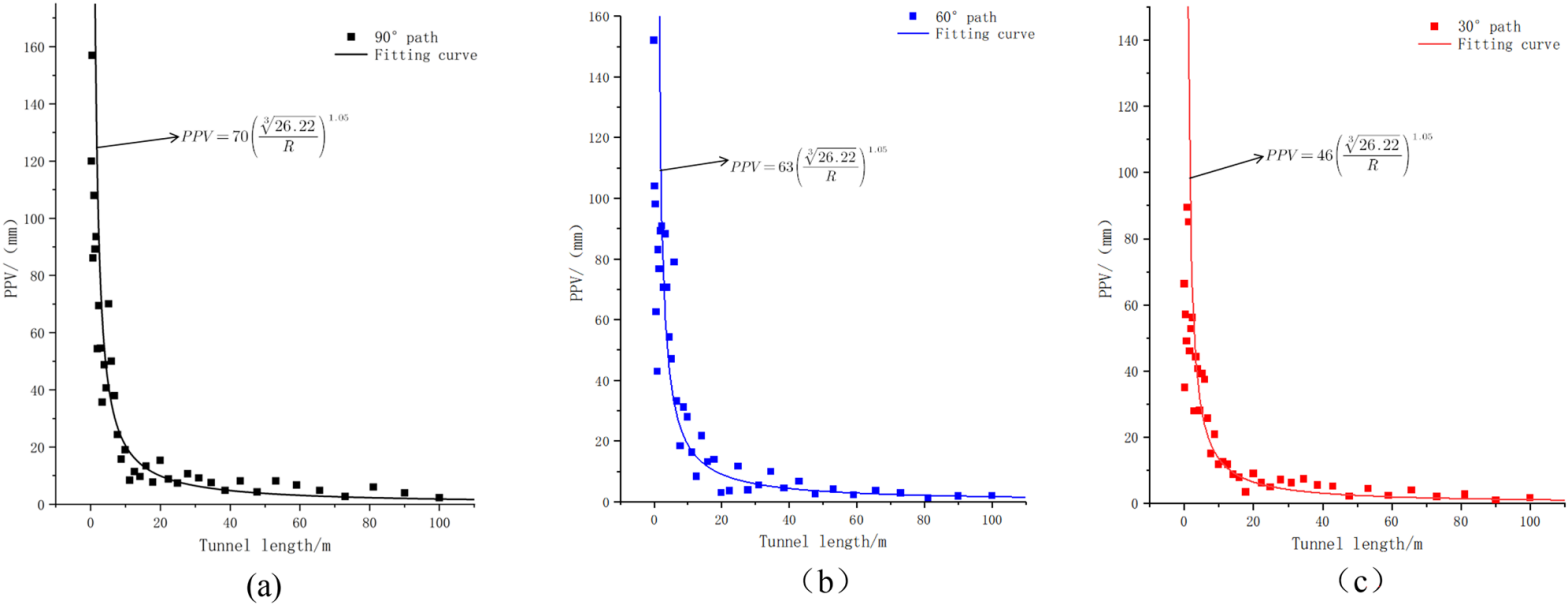
PPV fitting curves at different angles.

The Sadovsky formula is expressed as follows:

26 $$PPV = k\left( {\frac{{\sqrt[3]{Q}}}{R}} \right)^{\sigma }$$

where *Q* is the max. charge per delay (kg), From the volume and density of explosives, it can be seen that the mass of a single burst hole charge is 13.8 kg, and there are 19 burst holes, so *Q* = 26.22. *R* is the distance between the blast source and the monitoring point, *k* is a constant parameter related to the rock characteristics and geological conditions from the blasting point to the monitoring station, and *σ* is the explosive design parameters. Figure 14 shows the fitted curves of the PPV for the paths of the tunnel crown at angles of 90°, 60°, and 30°.

Figure 14 shows that the maximum PPV of the lining structure occurred when the tunnel crown angle was 90° during circular blasting. Therefore, by applying the data from Figure (a) to the specified values in the current Chinese blasting regulation “Blasting Safety Regulations,” the following calculations can be obtained: For a concrete age of 0–3 days, the safe distance is greater than 62.5–91.5 m. For a concrete age of 3–7 days, the safe distance is greater than 27.8–62.5 m. For a concrete age of 7–28 days, the safe distance is greater than 16.9–27.8 m. Table 8 compares the allowed PPVs for the new concrete specified in the regulation with the calculated results.

2. Considering the Sadovsky correction formula for different tunnel crown angles

**Table 8:** New concrete limit PPV and safety distance.

Age/d	0–3	3–7	7–28
Safe PPV/(cm/s)	1–3	3–7	7–12
Safe distance/m	62.5–91.5	27.8–62.5	16.9–27.8

Figure 14 shows that the geological parameter k in the fitted curve varies with the tunnel crown angle. This trend shows that as the tunnel crown angle decreases, the PPV also decreases. Therefore, during circular blasting in tunnels, the strength of the lining structure at the tunnel crown should be prioritized. Additional reinforcement should be applied to the top concrete to prevent stress failure caused by excessive PPV. The parameters of the Sadovsky formula for different angles are listed in Table 9.

**Table 9:** Formula parameter table.

Angle/(°)	*k*	*Q*/(kg)	*σ*
90°	70	19 × 1.38	1.05
60°	63		
30°	46		
0°	29		

**Figure 15:**
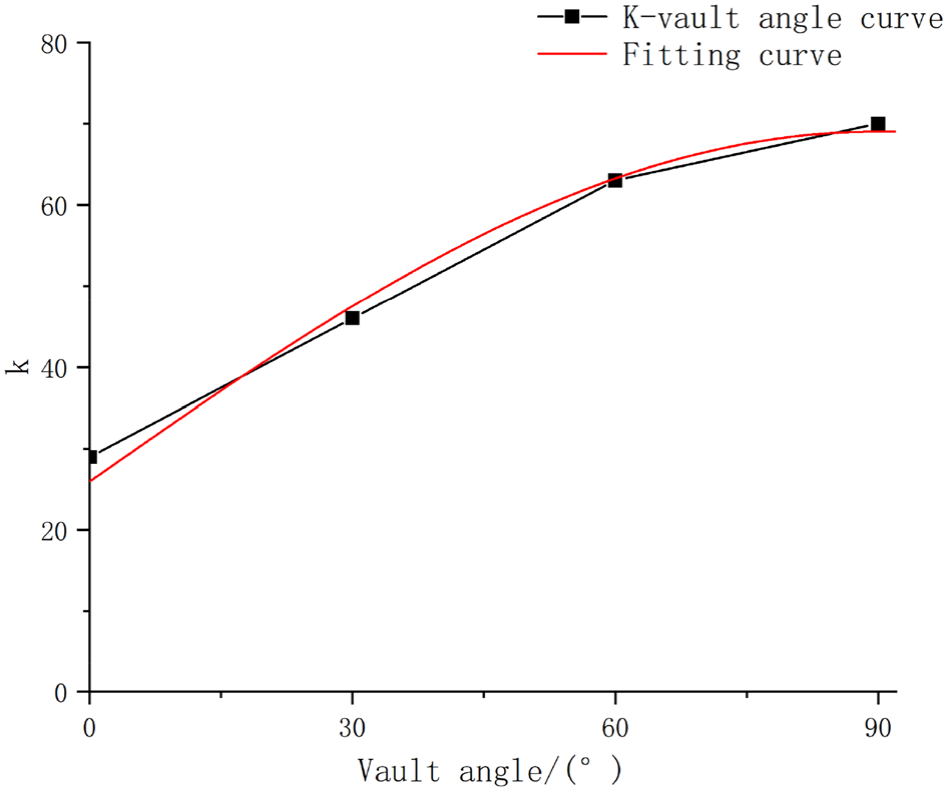
K-vault angle fitting curve.

The fitting equation in Fig. 14 reveals that, for smooth blasting, the magnitude of the PPV along the tunnel path increases with an increase in the tunnel crown angle. Furthermore, the increase is nonlinear. Therefore, to investigate the variation pattern of the PPV with the tunnel crown angle for smooth tunnels under circular blasting conditions, we plotted a k-tunnel crown angle curve, as shown in Fig. 15.

The geological parameter *k* of the Sadovsky formula was further fitted to obtain the Sadovsky correction formula considering the tunnel crown angle as follows:

27 $$k_{i} = 0.34k_{max} + 0.6k_{max} \times \sin \theta$$

28 $${\text{PPV}} = \left( {0.34 + 0.6\sin {\uptheta }} \right){\text{k}}_{{{\text{max}}}} \left( {\frac{{\sqrt[3]{Q}}}{R}} \right)^{1.05}$$

### Safety distance based on concrete tensile failure

**Figure 16:**
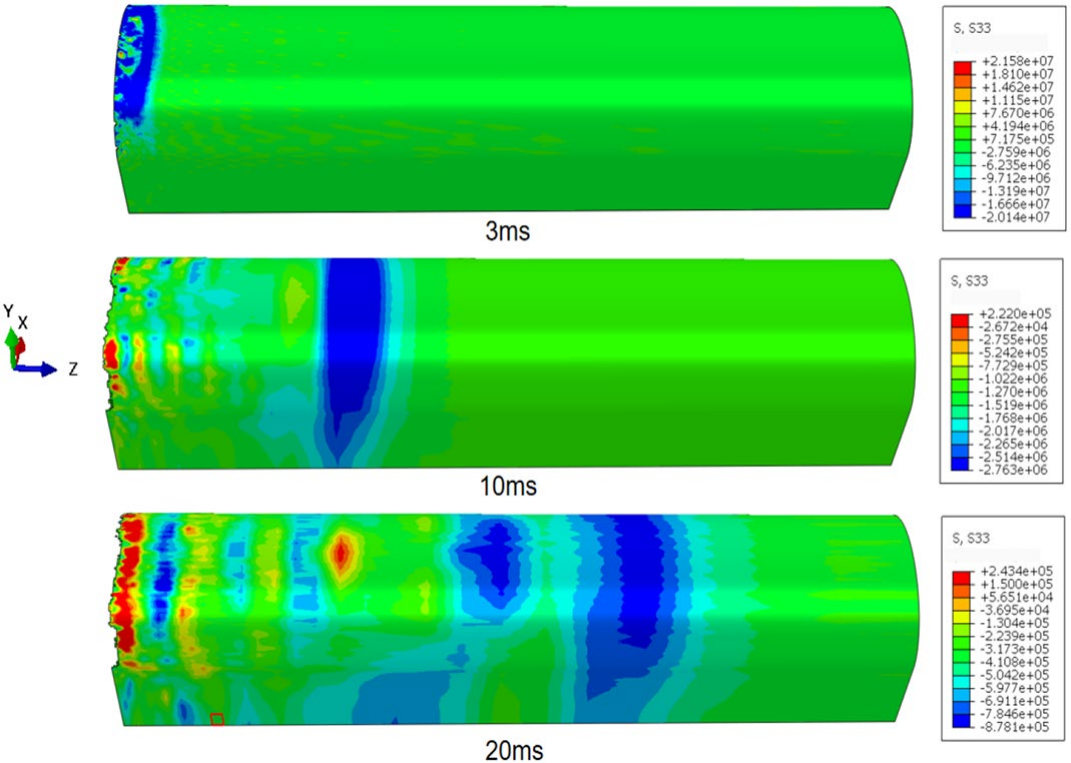
Tensile stress nephogram.

**Figure 17:**
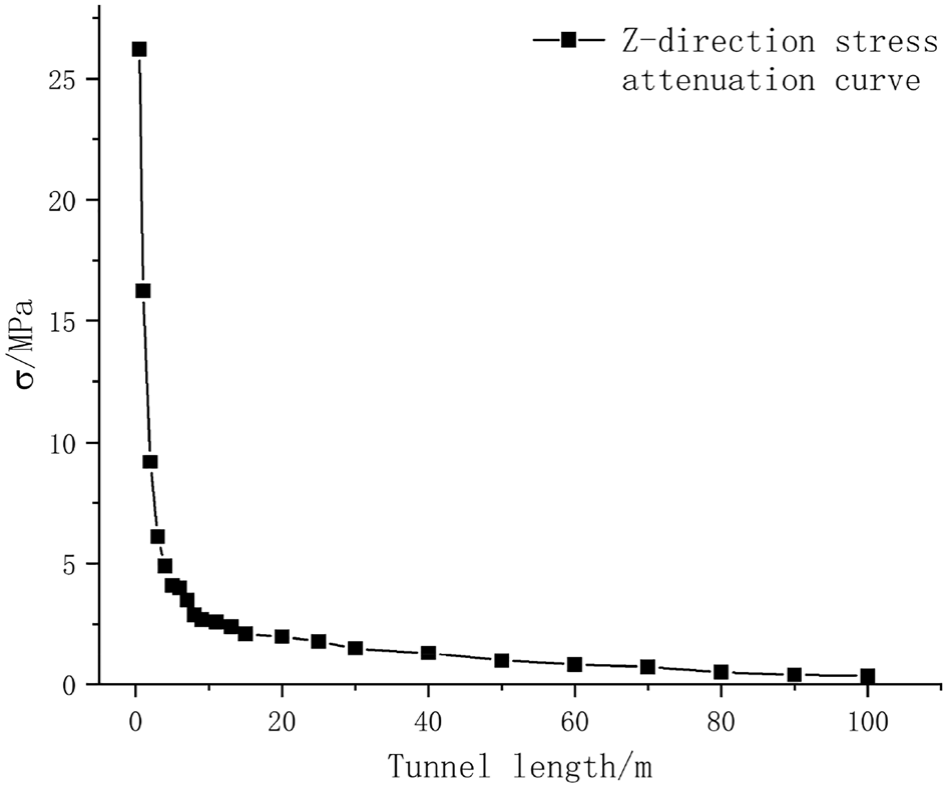
Peak stress change curve.

Figure 10 shows that the stress wave in the Z direction of the tunnel lining structure during blasting is much greater than the lead stress wave mentioned in “Stress wave transmission law” section. This stress wave in the Z-direction can lead to the tensile failure of the concrete structure. Therefore, this section focuses on studying the safe distance for the tensile failure of the tunnel lining structure under construction using the ring blasting method. The diffusion nephograms of the stress wave in the Z-direction at different times are shown in Fig. 16. The nephograms show that the stress wave attenuated during the propagation process, and the stress distribution at the crown was similar to that of the PPV, reaching its maximum at 90° from the crown. After the first stress wave generated by the explosion diffused, a negative stress wave in the Z-axis direction appeared in the range 0–5 m behind the tunnel face. Its propagation speed was lower, and its magnitude was significantly smaller than that of the first stress wave. In summary, the stress peak during the propagation of the first stress wave was considered as the stress variation curve, as shown in Fig. 17.

The dynamic tensile strength of the concrete at different ages can be derived using the following formula. Wang^31^ experimentally obtained the static tensile strength of C30 concrete. Under dynamic loading conditions, the tensile strength of concrete is expected to increase to varying degrees compared with that under static conditions. Under the action of blasting loads, the strain rate of the concrete structure is 17.3 to 21.3 s^−1^^32^. The dynamic tensile strength of concrete can be calculated using the strain rate and the formula^33^

29 $${\text{f}}_{{{\text{td}}}} = \left[ {1.95 - 3.32\left( {\frac{{1 - {\dot{\varepsilon }}^{1/8} }}{{2.2 + 3.2{\dot{\varepsilon }}^{1/8} }}} \right)} \right]f_{t0}$$

The dynamic tensile strengths of the C30 concrete at different ages were found to be 4.9, 5.4, and 7.8 MPa. By substituting these values into the peak stress variation curve shown in Fig. 15, the corresponding safety distances for different ages under the dynamic tensile strength standards were 4.0, 3.4, and 2.6 m, respectively. The relationship between tensile strength and safety distance is summarized in Table 10 below:

**Table 10:** Compressive strength of concrete.

Concrete age/d	7	14	28
Static compressive strength/MPa	2.3	2.5	3.6
Dynamic tensile strength/MPa	4.9	5.4	7.8
Safe distance/m	4.0	3.4	2.6

By comparison and analysis, we can conclude that the safety distance considering concrete tensile damage is much smaller than the safety distance considering PPV standards. Therefore, the impact of concrete tensile failure can be ignored during tunnel blasting, which is consistent with the conclusions of Yang^5^ through a theoretical analysis.

## Conclusion

This study determined the JH-2 model parameters for the surrounding rocks in practical constructions through simplified experiments and theoretical derivations. By combining the JH-2 models for concrete and granite and relying on typical large-section tunnel blasting projects, a quantitative analysis of the safety of lining structures during tunnel blasting was conducted using numerical calculation methods. The research findings are as follows:

- In ring blasting, owing to the different strength parameters of concrete and surrounding rocks, the bonded surface between the concrete and rock mass experiences advanced stress diffusion. In the simulated analysis in this project, the difference in advanced stress waves on both sides of the bonded surface did not cause damage to the bonded surface. The use of concrete with strength parameters similar to those of the surrounding rocks effectively reduces the occurrence of advanced stress diffusion.
- In ring blasting, the arch crown angle affects the PPV of the lining structure. The larger the angle of the arch crown, the greater the PPV. A Sadovsky correction formula considering the angle of the arch crown was derived. When constructing the tunnel linings, additional curing must be applied to the concrete at the top of the tunnel to increase the bonding strength of the bonded surfaces.
- Compared with the safety distance determined based on the PPV standards, the safety distance considering concrete tensile failure was approximately one-tenth that of the former. Therefore, during blasting construction, priority should be given to the damage to the concrete–rock mass-bonded surface caused by the PPV.

## Data Availability

The datasets generated and/or analysed during the current study are not publicly available due [REASON WHY DATA ARE NOT PUBLIC] but are available from the corresponding author on reasonable request.
